# Artificial intelligence in pediatric allergy research

**DOI:** 10.1007/s00431-024-05925-5

**Published:** 2024-12-21

**Authors:** Daniil Lisik, Rani Basna, Tai Dinh, Christian Hennig, Syed Ahmar Shah, Göran Wennergren, Emma Goksör, Bright I. Nwaru

**Affiliations:** 1 Krefting Research Centre, Institute of Medicine, Sahlgrenska Academy, University of Gothenburg, Box 424, 405 30 Gothenburg, Sweden; 2 Division of Geriatric Medicine, Department of Clinical Sciences in Malmö, Lund University, 214 28 Malmö, Sweden; 3 CMC University, No. 11, Duy Tan Street, Dich Vong Hau Ward, Cau Giay District, Hanoi, Vietnam; 4 The Kyoto College of Graduate Studies for Informatics, 7 Tanaka Monzencho, Sakyo Ward, Kyoto, Japan; 5 Department of Statistical Sciences “Paolo Fortunati”, University of Bologna, Bologna, Italy; 6 Usher Institute, University of Edinburgh, Edinburgh, UK; 7 Department of Paediatrics, Sahlgrenska Academy, University of Gothenburg, Gothenburg, Sweden; 8 Wallenberg Centre for Molecular and Translational Medicine, Institute of Medicine, University of Gothenburg, Gothenburg, Sweden

**Keywords:** Allergic rhinitis, Allergy, Asthma, Artificial intelligence, Atopic dermatitis, Childhood, Children, Eczema, Infants, Machine learning, Pediatrics, Teenagers, Wheezing

## Abstract

Atopic dermatitis, food allergy, allergic rhinitis, and asthma are among the most common diseases in childhood. They are heterogeneous diseases, can co-exist in their development, and manifest complex associations with other disorders and environmental and hereditary factors. Elucidating these intricacies by identifying clinically distinguishable groups and actionable risk factors will allow for better understanding of the diseases, which will enhance clinical management and benefit society and affected individuals and families. Artificial intelligence (AI) is a promising tool in this context, enabling discovery of meaningful patterns in complex data. Numerous studies within pediatric allergy have and continue to use AI, primarily to characterize disease endotypes/phenotypes and to develop models to predict future disease outcomes. However, most implementations have used relatively simplistic data from one source, such as questionnaires. In addition, methodological approaches and reporting are lacking. This review provides a practical hands-on guide for conducting AI-based studies in pediatric allergy, including (1) an introduction to essential AI concepts and techniques, (2) a blueprint for structuring analysis pipelines (from selection of variables to interpretation of results), and (3) an overview of common pitfalls and remedies. Furthermore, the state-of-the art in the implementation of AI in pediatric allergy research, as well as implications and future perspectives are discussed.

*Conclusion*: AI-based solutions will undoubtedly transform pediatric allergy research, as showcased by promising findings and innovative technical solutions, but to fully harness the potential, methodologically robust implementation of more advanced techniques on richer data will be needed.

**Table:** 

**What is Known:** •* Pediatric allergies are heterogeneous and common, inflicting substantial morbidity and societal costs.* •* The field of artificial intelligence is undergoing rapid development, with increasing implementation in various fields of medicine and research.*
**What is New:** •* Promising applications of AI in pediatric allergy have been reported, but implementation largely lags behind other fields, particularly in regard to use of advanced algorithms and non-tabular data. Furthermore, lacking reporting on computational approaches hampers evidence synthesis and critical appraisal.* •* Multi-center collaborations with multi-omics and rich unstructured data as well as utilization of deep learning algorithms are lacking and will likely provide the most impactful discoveries.*

**Supplementary Information:**

The online version contains supplementary material available at 10.1007/s00431-024-05925-5.

## Introduction

Artificial intelligence (AI) is defined as computer systems that are capable of performing tasks that typically require human intelligence. AI is arguably the most transformative technology of the modern age, and has revolutionized science in waves over the past decades [1, 2], from hard-coded rule-based systems, to machine learning (ML) algorithms that learn from data, and most recently generative AI that enable interactive synthesis and generation of text and images/audio [3, 4]. AI is fundamental to automate and optimize patient management [3], accelerate drug discovery [5], and democratize healthcare-related knowledge [6]. AI even holds potential as a digital assistant in the conceptualization of research and its communication [7].

Pediatric allergic diseases are heterogeneous and intricately interrelated [8]. In combination with increased availability of large-scale (bio)medical data, pediatric allergy research is a suitable context for AI-driven research [9, 10]. The high prevalence of pediatric allergies [11–14] also promises societal benefits from AI applications in clinical practice (e.g., decision support systems) and targeted preventive measures. However, challenges need to be met, including patient privacy, validity, generalizability, specificity, contextualization, bias, and explainability [10, 15, 16]. Furthermore, given the rapid development in AI, up-to-date methodological insight is vital, but most clinicians lack such training and many research groups do not include/co-operate with specialists [17]. This review is intended as a guide and reference for conducting AI-based research in pediatric allergy. It is composed of three sections: (1) introduction of relevant AI terminologies/concepts, techniques, common pitfalls and remedies, and a blueprint for structuring and reporting AI-based studies; (2) overview of studies implementing AI in pediatric allergy in unique and impactful ways; (3) a discussion of limitations and possibilities of AI in pediatric allergy.

## Brief background into the field of AI

### Machine learning

**Fig. 1:**
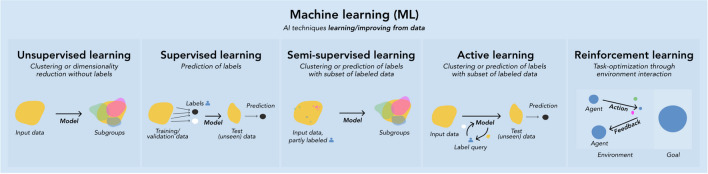
Subdomains of machine learning divided by learning mechanism

Machine learning (ML) is a subset of AI. As with other AI techniques, ML algorithms (models) mimic human intelligence by solving problems and performing tasks “intelligently”. In ML, however, there are no preprogrammed rules for how to perform tasks or solve problems; instead, these are derived from patterns that the models learn from data based on mathematical principles. The underlying objective is to measure and enhance specific tasks, e.g., identifying subgroups in a patient sample [18]. ML is commonly categorized by the mechanism by which it learns from data. Briefly, in supervised learning, the model learns patterns associated with provided labels, aiming to predict labels in new data (e.g., unseen patients with certain symptoms having a disease or not). Supervised learning can be divided into classification (categorical labels, e.g. asthma subtypes) and regression (continuous labels, e.g. lung function results). In unsupervised learning, the model does not involve labels, and instead “independently” explores distinct patterns (subgroups) within data (e.g. trajectories of eczema) onto which it provides label suggestions. Semi-supervised and active learning are useful on large data in which a small subset is labeled and for which labeling is difficult/time-consuming, and differ mainly by the mechanisms by which they assign labels to unlabeled data [19]. Finally, in reinforcement learning, a virtual artificial environment is created, within which a task (e.g. drug dosing to reduce complications while maximizing survival/recovery rates) is simulated by the model and optimized based on negative/positive feedback from the virtual artificial environment [20, 21] (Fig. 1).

Artificial neural networks (ANNs) are a subtype of ML conceptually inspired by the neuron structures in the brain. The basic unit consists of neurons, receiving input from and transmitting output to other neurons based on specific functions/criteria. ANNs are composed of at least one hidden (processes therein cannot be directly observed) neuron layer and an input and output layer. Deep neural networks (DNN) are ANNs with multiple hidden layers [22], applications of which are commonly referred to as deep learning (DL). Numerous neural network types have been developed for specific tasks, such as convolutional neural networks, suitable for image analyses [23], and recurrent neural networks, applicable in sequential data/trajectory analyses [24]. The model used must be thoughtfully selected, due to model assumptions regarding data, type(s) of patterns learned, and output interpretation. Summaries of common models are presented in Table 1. Many methods used today have a long history, originally stemming from fields such as statistics [25]. Due to the development of computing power, they can nowadays be used on large datasets and constitute standard components of the ML toolbox.

**Table 1:** Commonly used machine learning models

Model name^a^	Appropriate use-cases / assumptions of data	Advantages/strengths	Disadvantages/limitations	Examples in pediatric allergy / further reading
***Unsupervised learning***				
***k*-means** / k-means + + [26], (kernel) fuzzy c-means [27] etc	▪ Cross-sectional continuous data ▪ Assumes clusters to be homogeneous with similar within-cluster variation along all variables	▪ Implementations widely available, with variants for various types of data and clustering objectives, e.g., fuzzy c-means (probabilistic labeling) [28] ▪ Computationally efficient	▪ Unsuitable for categorical/mixed data ▪ Unsuitable if clusters have strongly different within-cluster variation ▪ Prioritizes within-cluster homogeneity over between-cluster separation	▪ Endotypes of seasonal AR based on cytokine patterns [29] ▪ Immunologic endotypes of AD in infants [30] ▪ Phenotypes of asthma based on demographics, comorbidities, and medication [31] ▪ Biomarker-based phenotypes of AD [32] ▪ Transcriptomic clusters of asthma [33]
***k*-medoids** / **partitioning around medoids (PAM)** / FastPAM / CLARA / CLARANS [34–37] / fuzzy k-medoids [38] etc	▪ Cross-sectional continuous or mixed data ▪ More flexible than *k*-means but still focuses on within-cluster homogeneity	▪ Relatively non-sensitive to outliers ▪ Can use any distance metric [39] ▪ Somewhat computationally costly, but high-performance variants are available, e.g., [34, 35, 40]) ▪ Implementations widely available, with various iterations for specific clustering objectives, e.g., with soft labeling [38]	▪ Spherical/convex clusters are more likely to be identified [41] ▪ DBSCAN and other methods may be more appropriate in case of very different cluster sizes, and if between-cluster separation is more important than within-cluster homogeneity [42]	▪ Longitudinal phenotypes of AR based on mHealth symptoms/medication data [43] ▪ Phenotypes of AR based on demographic, heredity, and clinical data [44] ▪ Phenotypes of eczema based on longitudinal disease patterns [45] ▪ Longitudinal phenotypes of wheezing [46, 47] ▪ Endotypes of asthma based on exhaled breath condensate [48]
**Hierarchical clustering** (**HCA**) / HCA on principal components (HCPC) [49] etc	▪ Cross-sectional continuous, categorical, or mixed data	▪ Accommodates any distance metric and a variety of linkage functions [50] ▪ Collinear/high-dimensional data can be managed automatically in HCPC [49], which combines feature extraction with HCA on the principal components ▪ Implementation widely available, as are various hierarchical methods with different focus, e.g., on homogeneity or separation, outliers etc. [51]	▪ Computationally costly with large data [50, 52] ▪ Meaningful clusters may only occur at low levels of the hierarchy, requiring potentially many clusters (some of which may just be outlier clusters) ▪ Clusters merged once cannot be separated again, which can result in suboptimal solutions	▪ Endotypes of rhinitis [53] ▪ Endotypes of seasonal AR [29] ▪ Phenotypes of AD based on allergic sensitization patterns [54] ▪ Phenotypes of asthma based on comorbidity, demographics, and asthma symptoms/lung function [55] ▪ Clusters of family adaptation to child’s FA [56] ▪ Clusters of asthma treatment outcome [57]
**Latent class analysis** (**LCA**) / longitudinal latent class analysis (LLCA)	▪ Longitudinal or cross-sectional categorical data	▪ Computationally efficient ▪ Informative by providing probability of assignment (soft clustering) ▪ Statistically principled approach to estimate number of clusters available ▪ Interpretability of original variables	▪ Inter-class heterogeneity is possible ▪ Compared to other methods used for trajectory analysis, performance may be lower [58]	▪ Trajectories of wheezing, rhinoconjunctivitis, and eczema symptoms [59] ▪ Grass/mite sensitization trajectoriess[60] ▪ Longitudinal phenotypes of FA and AD [61] ▪ Subtypes of AR based on comorbidity, heredity, and sensitization [62] ▪ Sensitization patterns [63]
**Growth mixture modeling (GMM)**	▪ Longitudinal data (continuous, but some implementations can handle categorical variables)	▪ Implementations widely available ▪ Allows for within-class variation ▪ Statistically principled approach to estimate number of clusters available	▪ More computationally demanding than e.g., LCGA [64]	▪ Trajectories of wheezing and allergic sensitization [65] ▪ Trajectories of allergic sensitization [66]
**Latent class growth analysis (LCGA) / group-based trajectory modelling (GBTM)**	▪ Longitudinal data (continuous, but some implementations can handle categorical data) ▪ Small sample or complex model with convergence issue [67]	▪ Implementations widely available ▪ High homogeneity due to no within-class variation allowed	▪ May necessitate larger number of classes due to no within-class variation [68], which may prove problematic if sample size is small	▪ Trajectories of asthma/wheezing based on dispensing data and hospital admissions [69] ▪ Trajectories of wheezing [70] ▪ Trajectories of early-onset rhinitis [71] ▪ Trajectories of eczema [72, 73]
***Supervised learning***				
***k*-nearest neighbors (*k*-NN)**	▪ Primarily for classification but may also be used for regression ▪ Varying degrees of noise, data size, and label numbers [74]	▪ One of the most widely used methods, with easy implementation, available in most statistical software ▪ Robust to outliers/noise [75]	▪ High computational demand on large datasets [76]	▪ Prediction of persistent asthma [77] ▪ Prediction of asthma diagnosis [78] ▪ Multi-omics based prediction of asthma [79] ▪ Prediction of asthma exacerbations based on blood markers, FeNO, and clinical characteristics [80]
**Support vector machine (SVM)**	▪ High-dimensional data (continuous, but categorical variables can be supported [81])	▪ Performs well on high-dimensional and complex predictor data	▪ Prone to overfitting, more so than many other supervised learning methods [77] ▪ Low explainability	▪ Prediction of asthma diagnosis [78, 82] ▪ Prediction of symptomatic peanut allergy based on microarray immunoassay [83] ▪ Prediction of AD based on transcriptome/microbiota data [84]
**Decision trees (DT)**	▪ Classification or regression ▪ Complex large data [85] of both continuous, categorical, or mixed nature	▪ Easily interpretable output	▪ Prone to overfitting ▪ Low accuracy compared to ensembles of DTs [86]	▪ Prediction of asthma diagnosis [78, 82] ▪ Prediction of hospitalization need for asthma exacerbation [87] ▪ Prediction of symptomatic peanut allergy based on microarray immunoassay [83]
**Random forests (RF)**	▪ Classification or regression ▪ Continuous, categorical or mixed data	▪ Relatively low risk of overfitting [88]	▪ Limited interpretability [88]	▪ Prediction of persistent asthma [77] ▪ Prediction of asthma diagnosis [78, 82] ▪ Prediction of hospitalization need for asthma exacerbation [87] ▪ Prediction of AD based on transcriptome/microbiota data [84] ▪ Prediction of asthma exacerbations based on AI stethoscope, parental reporting [89]
**Bayesian network**	▪ Continuous (although often discretized), categorical, or mixed data based on a probabilistic graphical model (directed acyclic graph)	▪ Adaptive by possibility to refine the network with new information [90] ▪ Capability of displaying and analyzing complex relationships [91] ▪ Accommodating missingness by utilization of all variables in model [90] ▪ Intuitive interpretation due to probabilistic labeling [92]	▪ Loss of information in discretization [90] ▪ High computational demand on large datasets [93]	▪ Prediction of asthma exacerbation [91] ▪ Prediction of response to short-acting bronchodilator medication [92] ▪ Metabolomic prediction of asthma [94] ▪ Prediction of eczema and asthma, respectively, based on SNP signatures [95]
**Naïve Bayes**	▪ Primarily for classification	▪ Needs relatively little training data [75] ▪ Computationally efficient [96]	▪ Strong assumptions of independence between the features [97] ▪ May need discretization of continuous variables [98]	▪ Prediction of OFC outcome [99] ▪ Prediction of asthma [78] ▪ Prediction of persistent asthma [77]
**Multilayer perceptron (MLP)**	▪ Complex high-dimensional continuous data	▪ Ability to learn complex patterns from high-dimensional big data	▪ May require large data for training, particularly if deep/complex network architecture [100]	▪ Prediction of asthma diagnosis [78] ▪ Multi-omics-based prediction of asthma [79]
**Extreme gradient boosting (XGBoost)**	▪ Classification or regression ▪ Continuous and categorical data [75] ▪ High-dimensional and/or sparse data [101]	▪ Improving accuracy through ensemble of weak prediction models ▪ Computationally efficacious [10]	▪ Performance issues may arise in imbalanced data [101] ▪ High memory usage	▪ Prediction of persistent asthma [77] ▪ Prediction of AD based on transcriptome/microbiota data [84]
**Adaptive boosting (Adaboost)/**	▪ Classification, but can be adapted for regression ▪ If crucial to boost performance of simple models through ensemble methods	▪ Combines multiple weak classifiers to create a strong classifier, improving accuracy ▪ Often achieves better performance with less tweaking of parameters compared to other complex models	▪ Sensitive to noisy data and outliers, which can lead to decreased performance if not handled properly ▪ Can overfit if the number and size of weak classifiers is not controlled	▪ Prediction of allergy [102] ▪ Allergy diagnosis framework [103] ▪ Asthma treatment outcome prediction [104]
**Logistic Regression (LR)**	▪ Primarily for binary (but can be extended to multiclass, e.g., with one-vs-rest strategy) classification ▪ Suitable for models where the outcome is a probability 0–1	▪ Simple implementation ▪ Efficient to train ▪ Well-understood and widely used ▪ Comparable performance to more advanced models in specific contexts of binary classification problems [105]	▪ Prone to underfitting when relationship between features and target is non-linear, unless feature engineering is applied ▪ Susceptible to overfitting with high-dimensional data if not regularized appropriately ▪ Assumes linear connection between dependent and independent variables, which can be limiting in complex scenarios [106]	▪ Prediction of house dust mite-induced allergy [107] ▪ Prediction of asthma persistence [77] ▪ Prediction of FA [108] ▪ Prediction of AD based on transcriptome and microbiota data [84]

The list is not intended to be comprehensive or cover all relevant/possible use-cases, but rather to provide an overview of common and promising algorithms. **Abbreviations.** *AD*, atopic dermatitis; *AI*, artificial intelligence; *AR*, allergic rhinitis; *FA*, food allergy; *FeNO*, fraction of exhaled nitric oxide; *N/A*, not available; *OFC*, oral food challenge; *SNP*, single nucleotide polymorphism

**Fig. 2:**
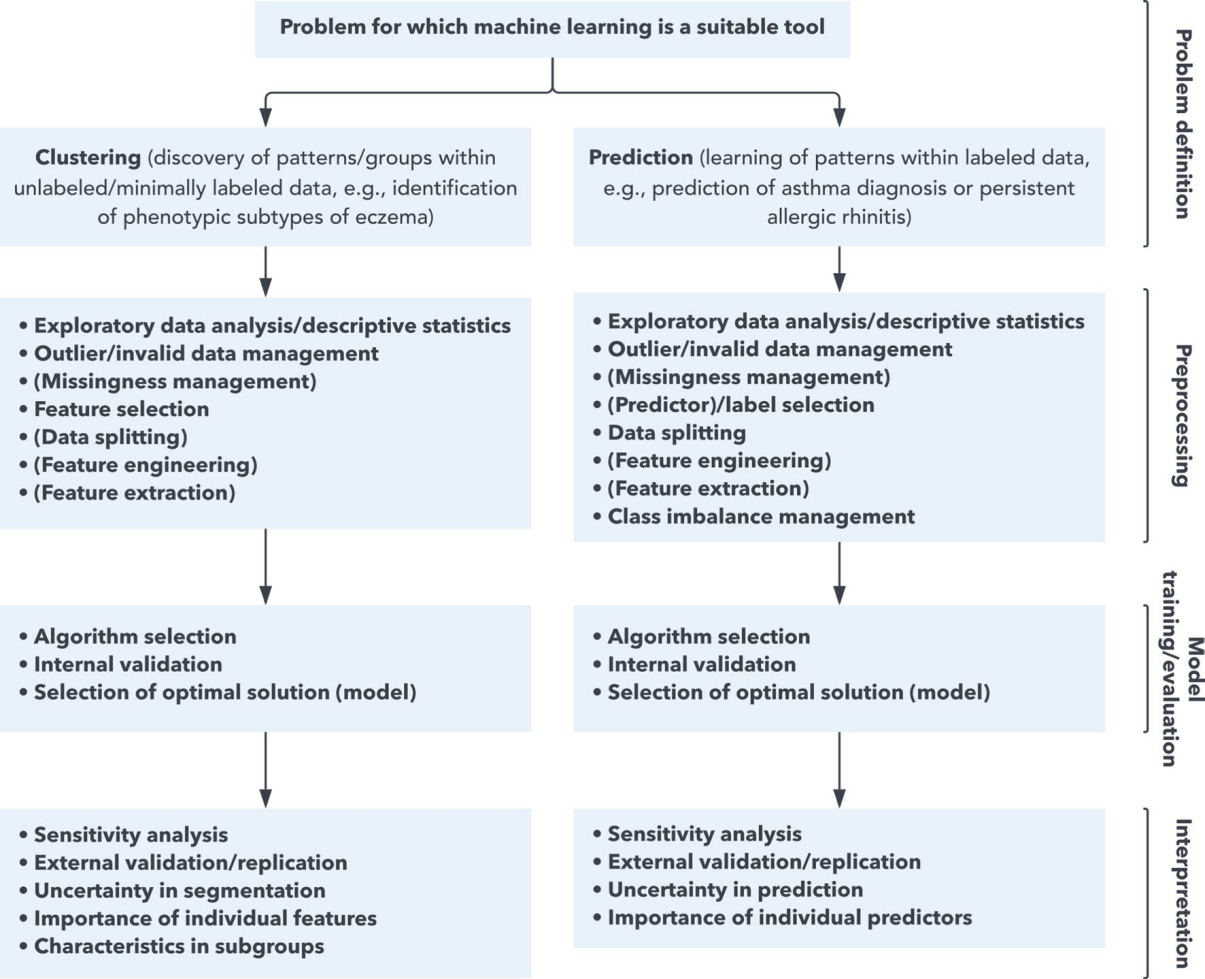
Recommended flowchart for building a machine learning pipeline

The following subsections describe terminology, techniques, and pitfalls (and remedies) for each step of an ML pipeline — from preprocessing to interpretation of results. A flowchart aimed to guide structuring of such pipelines is presented in Fig. 2.

A non-comprehensive general workflow with recommended steps to include in a study utilizing machine learning. In specific contexts, certain steps may not be needed or appropriate. Likewise, some studies may warrant specific steps not mentioned here. Items fully encapsulated in parentheses indicate that some machine learning models manage said issue “automatically” or performance evaluation may not be clear.

### Main steps of machine learning pipelines

#### Preprocessing

Preprocessing is often the most time-consuming step and heavily influences model performance/output. Exploratory data analysis typically precedes, to understand data distribution, patterns of missingness, and differences between the analyzed subsample and excluded subjects. Commonly, data need to be transformed to suitable forms (feature engineering), e.g., one-hot encoding categorical variables, log-transforming skewed data, and standardization/normalization of variables on different scales (e.g., height and income, where the latter could otherwise dominate by magnitude). In many datasets, redundant/non-informative/ “noisy” variables are present, which may increase run-time and hamper performance and interpretation, necessitating feature selection. Beyond manually selecting sensible variables, a simple example of feature selection is based on correlation matrices, in which correlated variables are dropped. A related technique (feature extraction) also reduces data dimensionality, by transforming variables into a lower-dimensional subspace while minimizing information loss. Principal component analysis is a simple feature extraction technique, also managing correlated variables. Some preprocessing techniques may be counterproductive, e.g., categorizing continuous variables, which should be avoided if possible to retain maximal differentiating information. See Table 2 for a summary of common preprocessing techniques.

**Table 2:** Common/recommended preprocessing steps/techniques

Preprocessing step	Substep or rationale/use-case	Techniques
**Exploratory data analysis (EDA) / descriptive statistics**	Provide an overview of patterns and potential imbalance in data. In case of stratified analyses (e.g., by sex) or subset analysis (e.g., those completing follow-up), comparison with table of characteristics and statistical tests for difference is useful	▪ Plots (ordinal/continuous data: histograms, nominal data: bar plots) ▪ Tables (for nearly any type of data; informative but should preferably be combined with visual presentations for ease)
**Outlier/invalid data management**	Outliers may be naturally occurring, but may also be due to measurement error, erroneous transfer of data etc. Removing outlier/invalid data is sensible (to improve representativeness and decrease impact of unusual measures) depending on the data and model/task at hand. Exploratory data analysis is needed to evaluate this. Importantly, over-removing outliers may reduce generalizability and increase the risk of overfitting [109]. If negative impact is suspected, sensitivity analysis can be performed to compare robustness/trends in the output. The ML models used also influence the need for removing outliers, as some are (more) robust to outliers. Invalid data management requires manual assessment with exploratory data analysis and domain-expertise	▪ Manual removal, e.g., of measures below/above a certain factor (commonly 1.5; 3 may be considered as *extreme* outliers) of interquartile ranges [IQR]. If assuming Gaussian distribution, removing measures outside of the 95th confidence interval may also be sensible ▪ Data-driven approaches (e.g., local outlier factor [LOF] [110]) ▪ For unsupervised learning, DBSCAN, HDBSCAN, and trimming approaches can facilitate identifying and handling noise in the data ▪ Model diagnostics can identify outliers after fitting a model; sometimes this is needed to see that an observation is outlying with respect to the modelled general pattern
**Missingness management**	The (likely) mechanism of missingness [111] must be evaluated to decide a suitable approach to substitute missing value or exclude subjects with (certain degrees of) missingness. Exploratory data analysis is key in this evaluation. Multiple imputation is generally preferred, to reduce the uncertainty in individual substitutions by producing several imputed datasets. The minimum number of imputed datasets is debated, with various rules of thumb [112]. There are also data-driven methods to decide the number of datasets needed for a specified precision of standard error [112]. Selecting an imputation method requires attention to data type (e.g., cross-sectional or longitudinal [113]) and association between variables [114]. In many cases, the analysis should be performed in each imputed dataset and pooled [115]	▪ Complete case analysis (either as main analysis or sensitivity analysis) ▪ Available case analysis (similar to the above, but dynamically select subjects with sufficient data for each individual analysis) [111] ▪ Imputation (e.g., multiple imputation) [113, 114, 116, 117]
**Feature/predictor/label selection**	In some datasets, particularly high-dimensional data, there may be irrelevant/non-informative/ “noisy” variables. While it is important to consider all variables, the inclusion of e.g., variables that are overlapping to a high degree clinically may decrease model performance, cause overfitting, and complicating interpretation	▪ Literature/expert-based selection, based on experience or literature ▪ Data-driven methods [118]: - Recursive feature elimination (RFE) [78] - Sequential feature algorithms [119, 120] - LASSO (combines variable selection and regularization to improve prediction accuracy and model interpretability [121]) - Tree-based approaches (e.g., decision trees, random forests) [118], and wrapper around said approaches, e.g., Boruta’s algorithm
**Data splitting**	To reduce overfitting, data typically needs to be split into a training set (for learning patterns), and a validation set (for performance assessment, to guide hyperparameter fine-tuning). Ideally, a third test set, agnostic of the preceding steps, is useful to assess the model performance on unseen data. In supervised learning, results from several approaches can be compared on a data subset not used for tuning; in unsupervised learning performance measurement is less obvious	▪ Cross-validation (*k-*fold validation, leave-one-out [LOO] etc.) [122] ▪ Bootstrapping ▪ Stability exploration in unsupervised learning
**Feature engineering**	**Feature scaling** Useful in continuous data and varying scale between features (e.g., height and annual income, the latter often on scales many times larger). Models (e.g., *k*-nearest neighbors) relying on distance metrics [123] perform better following feature scaling, as sheer magnitude of certain variable otherwise overinfluences the algorithm. Feature scaling should be performed after data splitting, as the sets otherwise introduce information to each other	▪ Normalization (rescales data to [typically] range 0–1; sensitive to outliers, useful when scale of variables are of higher importance, e.g., neural networks) [124] ▪ Standardization (rescales data to mean 0 and standard deviation 1; more robust to outliers and generally recommended for linear models, e.g., support vector machines) [124]
	**Discretizing/continuizing continuous/discrete variables** Discretizing converts continuous data into categorical bins, simplifying modeling, handling non-linear relationships, and improving interpretability by mitigating the effects of outliers. However, it causes information loss, and this is often a reason to avoid it. In contrast, continuizing converts categorical or ordinal data into continuous formats, capturing ordinal relationships and enhancing performance for algorithms that prefer numerical inputs, although this may not be feasible at times	▪ Entropy-MDL discretization ▪ Equal frequency discretization ▪ Equal width discretization ▪ One-hot encoding ▪ Frequency or mean encoding
**Feature extraction**	In addition to feature selection, dimensionality can also be reduced by creating new features that aggregate information in the original features. Many algorithms perform suboptimally with high-dimensional data (“curse of dimensionality”) [125]. In such cases, dimensionality reduction can be applied to reduce runtime and improve performance	▪ Linear techniques (e.g., principal component analysis [PCA; continuous data], multiple correspondence analysis [MCA; categorical data], MFA/FAMD [mixed data]; perform feature scaling prior to this step [126]) ▪ Non-linear techniques (e.g., autoencoders, t-SNE, UMAP) ▪ Subject-matter knowledge to define informative indexes summarizing several features
**Class imbalance management**	In supervised learning, there may be substantial imbalance of labels among the subjects. This imbalance can reduce the performance of the model	▪ Oversampling of minority class subjects (e.g., adaptive synthetic sampling approach [ADASYN] [127]) and/or undersampling of majority class subjects (e.g., by random exclusion)

The table describe common steps taken during preprocessing. The list is intended as a guide with steps presented in consecutive order as they should be performed, but it is not meant to be comprehensive or universally applicable. It is recommended, particularly for unique applications, to evaluate previous similar implementations or relevant technical literature.

#### Model training/evaluation

As per the “no free lunch” theorem [128], no model is ideal for all contexts. Thus, multiple models should be evaluated, the choice of which depends on the (1) nature of the data (density/distribution; tabular/non-tabular; numerical, categorical, or mixed; presence of outliers/noise etc.), and (2) task (relevant learning mechanism, desired utility/interpretability of output etc.). See Table 1 for a summary of common methods (additional methods are described in Table S1). Furthermore, it is important to accommodate differing and clinically influential factors (such as sex and age) of allergies and their presentation and outcomes. An AI application may otherwise produce biased and non-generalizable output when such factors are not accounted for [129]. Such unfairness in AI can be addressed using various approaches, e.g., by so-called “active fairness”, which essentially involves incorporating important “sensitive” attributes into the algorithm [130].

An important issue in cluster analysis is that there is no unique definition of an “optimal”/ “true” clustering. Most datasets allow for different reasonable clustering according to different criteria, e.g., requiring clusters to be homogeneous (low within-cluster distances), or making cluster separation the dominating criterion (which may lead to heterogeneous clusters if no gap separates dissimilar observations/subjects). It may also be reasonable to make model assumptions (e.g. Gaussian). These choices cannot be made from data alone; rather, they need to be made with the clustering requirements in mind [51, 131]. Ensemble clustering provides an alternative approach, in which clustering output from several algorithms are run through a consensus function to derive a final solution [132], and can be attractive in difficulty of deciding what kind of clusters are sought after. It must be noted, however, that ensemble clustering does not necessarily provide a more “correct” solution, as clustering algorithms are based on different concepts of what an optimal cluster is; this discrepancy is similar to meta-analysis, in which studies are often too heterogeneous for pooling. The corresponding subdomain in supervised learning (ensemble learning) [88, 133–135] is more often appropriate, as the different learners/models aim to solve the same underlying problem.

In unsupervised clustering, selecting a distance measure (i.e., measure of similarity/dissimilarity between subjects) is of importance, as it heavily influences evaluation metrics [50, 51, 136–138]. Another crucial issue in clustering is the choice of the number of clusters. In many situations the data do not determine an unambiguous number of clusters; rather, this will depend on the “granularity” of the clustering required for the application. Lower numbers of clusters are often easier to handle, but often larger numbers of clusters can improve the model fit. Several indices can be used for determining the number of clusters, but they commonly give conflicting results [51, 139]. Clinical interpretation is central in weighing between different alternatives.

Overfitting occurs when a model is overly specific to the training data, thus not generalizing well to unseen data. The risk of overfitting increases with model complexity and the number of variables in relation to the sample size [140]. Overfitting may also result from improperly separating training and validation data [10]. A common approach to combat overfitting is splitting data into: (1) training set (for training the model); (2) validation set (for fine-tuning); (3) test set (for performance evaluation) [122, 141, 142]. In neural networks, dropout regularization is a common technique to reduce the risk of overfitting. Underfitting is the opposite issue: the complexity of the data is not reflected in the model, e.g., too few variables or insufficient/non-representative training sample, and the performance is suboptimal on both training and unseen data [140, 143, 144].

Fine-tuning of hyperparameters (model settings) is generally necessary to optimize performance, typically done by comparing performance using different hyperparameters. A common technique is grid search (brute force search of all possible hyperparameter settings) [145], the practicality of which is limited in contexts with many possible settings. Random search is a compromise in such cases. Optuna [146], a hyperparameter optimization framework, and Bayesian optimization [147] are additional alternatives. The metrics assessed in training vary depending on the analysis aim(s), learning mechanism, and data, but should include multiple [148] metrics, e.g., accuracy, recall etc. (supervised learning) or intra-cluster separation, inter-cluster homogeneity etc. (unsupervised learning). In cluster analysis, some choices (e.g., dissimilarity measure) should aim at valid representation of the data within the context rather than optimizing cluster validity measures, as the meaning of criteria such as between-cluster separation and ultimately the clustering itself relies on the dissimilarity measure. Beyond optimizing a model on specific data, it is also valuable to stress-test the application under different conditions, such as assessing the stability of the algorithm with introduction of noise to the data, or by comparing the output of multiple algorithms. A summary of common evaluation approaches is presented in Table 3.

**Table 3:** Common/recommended model evaluation techniques

Technique	Rationale/use-case	Examples of implementation/approaches
*Unsupervised learning*		
Robustness	Unsupervised learning solutions robust to noise are reasonably (more) generalizable and less overfit to the trained-on data	▪ Bootstrapping ▪ Sensitivity analysis
Clinical interpretability	In unsupervised learning, algorithms may derive clusters/trajectories that differ by statistical measures, but are so similar that they are not clinically distinguishable. Assessing clinically the characteristics of subgroups is highly relevant. In supervised learning, this objective is less obvious, but clinically sensible predictor variables driving predictions most strongly could indicate a sensible/practically useful solution	▪ Tables and other textual representation ▪ Figures, e.g., heatmaps, line plots etc
Homogeneity within clusters and dissimilarity across clusters	The classical definition of well-defined clusters/trajectories, i.e,. that the subjects within the derived subgroups are similar to each other and dissimilar to subjects in the other subgroups). From a clinical perspective, this may also be done with e.g., heatmaps	▪ Silhouette score ▪ Calinski-Harabasz score ▪ Davies-Bouldin score
Model fit / complexity	Statistical measures of how well a model fits the observed data (with varying penalty for complexity of the solution) provides an easily interpretable metric for selecting the optimal model in a data-driven manner	▪ Akaike information criterion (AIC; commonly suggests more complex solutions and larger number of subgroups) ▪ Bayesian information criterion (BIC; in contrast to AIC emphasizes simpler solutions [149])
*Supervised learning*		
Performance metrics	Various measures are available to assess how accurately the model predicts based on new input data, varying widely depending on the learning task and data at hand. Ideally, multiple metrics need to be evaluated to assess model performance, some being more suitable in specific types of prediction models [148, 150, 151]	▪ R^2^, adjusted R^2^, MAE, RMSE, MAPE etc. (regression) ▪ MCC, F_1_ score, Cohen’s kappa, Brier score, AUROC, AUPRC, log-loss, accuracy, recall, specificity, NPV/PPV etc. (classification)
Validation curve(s)	Diagnostic tool to assess the performance of a model in relation to hyperparameter settings (for example number of trees in a random forest model). More specifically, this can be used to e.g., evaluate hyperparameters that influences the model’s tendency to overfit/underfit	▪ Plot of performance metric on y-axis and hyperparameter on x-axis

The table describe common techniques used in model evaluation. The list is not meant to be comprehensive or universally applicable. It is recommended, particularly for unique applications, to evaluate previous similar implementations or relevant technical literature.

#### Interpretation

Models differ by assumptions and statistical methods and thus necessitate appropriate interpretation of results. As an example, in hard clustering algorithms (e.g., *k*-means), each subject is assigned to one cluster; however, it is possible that some subjects are not similar to subjects within their cluster, necessitating cautious conclusions about cluster homogeneity. Multiple techniques can be used to assess outliers, e.g. heatmaps [47]. A substantial part of interpreting the performance of a model is to elucidate determinant factors. SHapley Additive exPlanations (SHAP) [152] is a common tool to visualize the contribution/importance of each variable for prediction or clustering. Finally, externally validating the results in a comparable independent cohort is useful to evaluate model robustness and generalizability. A list of common interpretation approaches are shown in Table 4.

**Table 4:** Common/recommended approaches for interpreting model results

Approach	Rationale/use-case	Examples
Replication with independent data (external validation)	Deriving comparable results from a model in an external (independent) cohort/sample of individuals indicates that the patterns learned by the model are (relatively) generalizable. Caution is needed in interpretating such results, however, as it may be that the underlying characteristics are very similar in the external data, or conversely, very dissimilar, which complicates interpretation	▪ Re-running analysis in independent external cohort/population
Relation with external information	Mostly applicable in unsupervised learning. If derived subgroups indeed represent clinically distinct entities, it may be that these differ meaningfully in aspects not characterized in the model. For example, if clusters based solely on skin prick test results are composed of children with very similar sociodemographic background factors, it increases the probability that there may be non-random and clinically meaningful pathophysiological differences between the clusters	▪ Tables/plots of characteristics (with appropriate statistical significance tests) ▪ Figures, e.g., spider plots, bar plots etc
Explanation of feature importance	Machine learning models, particularly with higher degree of complexity, are not directly interpretable based on the output. To get a sense of the attributes and patterns that influenced the model the most, multiple techniques are available to visually/numerically present the most important parts of the data according to the model. Individual methods may or may not be applicable in specific models/contexts	▪ Class Activation Maps (CAM) [158] ▪ Local Interpretable Model-agnostic Explanations (LIME) [159] ▪ SHapley Additive exPlanations (SHAP) [152] ▪ Mean decreased Gini value [160]
Subgroup homogeneity	In unsupervised learning, the derived subgroups may have within-subgroup heterogeneity impacting interpretation of recognizability in the clinical setting, subsequent association analyses etc. For this reason, it is useful to present (indirect) measures of homogeneity/heterogeneity within derived classes. For trajectory analyses, the 95% confidence interval (95%CI) around the predictor prevalences/probabilities may indicate the degree of variation within trajectories. Tabular data may provide similar information as well	▪ Heatmaps [45–47] ▪ Tables/plots of characteristics (with 95%CIs)

The table describes common techniques used in model evaluation. The list is not meant to be comprehensive or universally applicable. It is recommended, particularly for unique applications, to evaluate previous similar implementations or relevant technical literature.

### Practical aspects of implementation

Software necessary for implementing AI-based applications are widely available, often through free open-source packages for Python and R statistical software. However, the major hurdle often lies in hardware and technical know-how. In terms of hardware, most “shallow” algorithms, as well as DL with small/moderate-sized data, can be performed on regular modern laptops/computers. For advanced modelling or analysis of large data, the use of dedicated hardware is often needed, in which case it is crucial to ascertain that sensitive data are not accessible by unauthorized parties. Although computational skills are much more time-consuming to develop, there are many resources aimed at lowering the barrier to implementing AI, e.g., low-code/no-code platforms/tools for performing AI-based analyses, such as MLpronto [153], Orange Data Mining, KNIME, as well as various online courses and certifications in AI for clinicians [154]. While everyone in a healthcare team does not necessarily need to learn how to program or develop in-depth knowledge about machine learning, it is important that colleagues have at least a basic understanding of fundamental concepts, to efficiently discuss AI implementations.

### Ethical considerations and reporting

Beyond understanding of technical intricacies, AI-based research in pediatric allergy necessitates particular attention to ethical aspects, e.g., pertaining to data protection, as AI algorithms are often fed vast amounts of sensitive information. Frameworks guiding safe inclusion of pediatric data in AI-based research include ACCEPT-AI [129].

Detailed reporting from all steps of the pipeline is crucial for critical appraisal and reproducibility/synthesis. For example, as allergic diseases demonstrate heterogeneous developmental patterns across age, it is essential to clearly report on age (and variation thereof) in the study population. Multiple checklists have been developed, e.g., Transparent Reporting of a multivariable prediction model for Individual Prognosis or Diagnosis (TRIPOD + AI) [155] for prediction models and Guidelines for Reporting on Latent Trajectory Studies (GRoLTS) [156] for trajectory analyses, although well-established equivalents are lacking in many areas, e.g. cluster analysis. While items essential to report vary depending on analysis and data, a general guideline is provided in Table 5. Reporting recommendations of AI-based clinical studies have also been proposed [157]. Additional guidelines can be found at the EQUATOR (Enhancing the QUAlity and Transparency Of health Research) Network (https://www.equator-network.org/).

**Table 5:** Recommended elements to report in machine learning-based studies

Step	Rationale/general description
Preprocessing	▪ **Exploratory data analysis/descriptive statistics** **- Cohort characteristics:** Describe in detail the used cohort/sample. Present the initial participation rate and relevant background factors for evaluation of generalizability and representability **- Analysis subset characteristics and comparison:** If a subset of the cohort/sample was used for the analysis, compare background factors between the full cohort/sample and the subset **- Correlation analysis:** Report correlations between variables (e.g., through a correlation matrix). Understanding these relationships can guide feature engineering and model selection, as well as reveal potential (multi)collinearity issues **- Missingness:** Visualization of the degree of missingness (across subjects and variables, preferably with a measure of patterns across missingness between variables) provides useful information as well ▪ **Outlier/invalid data management:** Describe the presence/degree of outliers and/or data deemed invalid, and if any processing of these was performed. Visualization is particularly useful, e.g., with simple box plots. If possible, provide code/syntax (in a repository) ▪ **Management of missingness:** Visualize/tabulate missingness and patterns thereof. Provide the rationale for using a particular imputation algorithm or other approach, including details (preferably including plots) on evaluation/validation of the imputation. If possible, provide code/syntax (in a repository) ▪ **Feature selection:** Provide explicit rationale for the used variables. Ideally, add a table (in the supplementary material) where the reason(s) for inclusion/exclusion for each variable that could potentially have been of relevance are listed. Importantly, feature selection processes should be described in detail, including narrative summary and output of data-driven approaches and tables. If data-driven methods were used, provide code/syntax (in a repository) ▪ **Feature scaling:** Report if the variables were inputted as-is or if any scaling was performed (preferably providing code/syntax) ▪ **Dimensionality reduction**: Report the tools and hyperparameters/settings used (preferably providing the actual code/syntax), together with details on the percentage of variance explained in the reduced subspace, loss, or other relevant information to assess the performance/representability of the new data
Model training / evaluation	▪ **Algorithm selection:** Describe the rationale for the selection of models. Preferably, select at least two models to assess the robustness of the chosen solution ▪ **Model implementation:** Explain in detail how the model(s) were implemented, which hyperparameter settings were tested and the underlying rationale. Preferably, provide the actual implementation code/syntax (in a repository) ▪ **Model evaluation:** Provide a detailed log of the model(s) with different hyperparameters, so as to make the selection of the optimal solution transparent and clear for the reader. For example, if a cluster analysis was performed and 3–5 cluster-solutions were assessed as the top 3 models, provide clinical characteristics and evaluation metrics for at least these (but preferably *all* tested solutions)
Interpretation	▪ **Characteristics:** Most relevant for unsupervised analyses. Provide rich details on the subgroups, including on parameters included and *not* included in the model (e.g., background factors, comorbidities, sociodemographic factors etc.) ▪ **Influencing factors/explanation of the model:** Provide as much detail as possible on how the model derived its output, e.g., feature importance ▪ **Uncertainty in findings:** Describe the uncertainty in the model (e.g., 95% confidence intervals of the predictions or subject characteristics) ▪ **External validation:** Provide an analysis of the generalizability of the results, preferably by externally validating the model in a different cohort/sample ▪ **Limitations:** Could the analyses have been done differently in an optimal setting? Transparently describe challenges and drawbacks/compromises

## Implementation in clinical practice and research

Implementation of AI across disciplines varies massively. For example, across United States Food and Drug Administration-approved medical AI devices, a majority have been developed for radiology, while none are listed under allergy [161]. Despite this, the need is paramount and the possibilities evident, given the availability of rich data and computational power, as well as rapid development of algorithms. While applications in clinical practice are largely absent, the utilization of AI within research is more established. In the following subsections, promising/impactful AI-based studies in pediatric allergy are summarized.

### Diagnosis of disease

In a study by Kothalawala et al., models were evaluated for predicting asthma at 10 years based on hospital records, clinical assessments, and questionnaire responses from multiple time points. Notably, just a few predictors (cough, atopy, and wheeze) contributed substantially [78]. He et al., conversely, focused on predicting asthma at 5 years, reporting limited predictive power of markers from infancy, with earliest reliable model being based on data at age 3 years. The authors also noted a clear progression of feature importance at different ages [162]. Overall, substantial heterogeneity is present across prediction models of asthma, and generalizability is moderate, rendering clinical implications unclear [77, 163]. This uncertainty is furthered by reports indicating comparable performance to regression models [164]. Sophisticated AI methods are demanding to implement; thus, they are rare and may not live up to their potential. Although prediction models using multi-omics data are scanty, there are indications that specific omics combination provide superior performance [79, 165]. Prediction models have also been applied in diagnosis of atopic dermatitis (AD) [84], allergic rhinitis (AR) [166], and more specific diagnostic labels, e.g. oral food challenge positivity to specific foods [99, 167]. Overall, these studies demonstrate applicability of various predictors and meaningful results, but also substantial heterogeneity and limited clinical utility due to lacking methodological reporting and impracticality of synthesis/comparisons.

### Subtyping of disease

Subtyping can be performed on cross-sectional (cluster analysis) or repeated measures (trajectory analysis). In a study by Havstad et al. [63], a cluster analysis of sensitization patterns, found four clusters, which were more strongly associated to AD and wheeze/asthma compared to any atopy. Studies focused on endotypic markers have also exemplified the heterogeneity of allergy, e.g., a study by Malizia et al. [29], in which three inflammatory patterns of seasonal AR were identified based on cytokine patterns. Longitudinal disease patterns have commonly been derived from binary (presence/absence) markers, and although clinically meaningful patterns have been identified, e.g. wheezing trajectories associated with (actionable) risk factors [168] and outcomes, such as allergic disease/sensitization in adulthood [169], important disease characteristics are omitted. Some studies have included severity measures, e.g., by Mulick et al. on eczema trajectories [170]. Given the reported uncertainty/within-class heterogeneity with latent class analysis [46, 171], a common algorithm in trajectory analysis, alternative methods, based on cluster analysis of longitudinal data condensed into characteristics such as onset-age, number of episodes with symptoms etc., as implemented by Haider et al., have contributed with homogeneous trajectories of eczema [45] and wheezing [46, 47]. Studies incorporating data from multiple sources, such as parental report and prescribed medication/physician assessment [172–174], have further contributed with distinct subgroups and highlighted the complexity underlying pediatric allergies and utility of multi-dimensional characterization.

### Management of disease

Successful management can be defined in various ways, including survival, treatment adherence, and remission. Typically, the same models as for diagnosis prediction are applicable. Tailored sequential models have shown promising accuracy in predicting adherence to subcutaneous immunotherapy in AR treatment [175]. Disease progress can also be modelled as trajectories. In a study by Belhassen et al. [176], trajectories based on use of inhaled corticosteroids before and after asthma-related hospitalization were derived and related to, among others, sociodemographic factors, which may provide guidance in individualized treatment. DL image analysis of eczema lesions [177] coupled with a mobile app has shown promising results in e.g., patient-oriented eczema measures [178], although large-scale studies are needed to validate such findings, and engagement rates need to improve. Similarly, an AI-assisted clinical support was evaluated in asthma management, showing comparable asthma exacerbation rates as with regular care, albeit reducing administrative burden [179], indicating limitations in clinical decision-making and potential in streamlining time-consuming tasks.

### Outcome prediction

Models for outcome prediction, typically architecturally equivalent to diagnosis prediction models, have been used in various settings, from relatively simplistic contexts and predictor sets, such as symptom and treatment recordings of eczema, aiming to predict future severity [180], to large-scale medical record data to predict asthma persistence [77]. In general, despite incremental improvement, even models incorporating > 100 predictors and large study samples are not yet fully refined for broad clinical implementation [181]. Associated health outcomes, such as suicidality in adolescents with AR, have also been investigated, adding important contribution to our understanding of the complex multi-dimensional nature and implication of pediatric allergy [182]. DL implementations are scanty [183].

## Limitations and future perspectives

Advanced AI is data hungry [184], and data are limited in many contexts. Possible solutions include data augmentation, active learning, and transfer learning [185]. This challenge is exacerbated by data protection laws limiting data sharing, particularly within the European Union. Non-disclosive distributed learning frameworks may facilitate collaboration, while large language models may aid in tedious standardization/harmonization. As an example, DataSHIELD is a freely available open-source framework for co-analysis of individual-level data, which allows original data to remain unseen by other entities [186]. Broad multi-disciplinary collaboration may also facilitate incorporation of multi-omics data, and ultimately aid in streamlining AI training through increased concentration of relevant expertise. National and international consortia require substantial administrative efforts, particularly at the early stage, but are increasingly needed to meet the demands of data and context-specific expertise.

Explainability is another pertinent issue, which has largely persisted, particularly for advanced algorithms [187–189], and which may introduce elusive biases. Increased involvement of patient-representative groups is key to continuously safe-guard and appropriately adjust models. Decision-makers are also often uninformed of technical specifics, potentials, and limitations of AI applications, resulting in unequal distribution of funding. Increased familiarity with AI is needed, as are well-established guidelines and easy-to-use frameworks for conducting analyses in a reproducible and appropriate fashion. These characteristics are largely lacking in the extant AI-based studies in pediatric allergy. Furthermore, there are important gaps in the literature. For example, few studies are focused on food allergy and allergic rhinitis in comparison to eczema and particularly asthma. It is likely that the most impactful discoveries will be made using DL approaches with large-scale multi-omics data (including wearables and other accessible/non-invasive gadgets) as well as nationwide/multi-national register data with cross-linkage, in which input is as unfiltered and comprehensive as possible. This will maximally harness the computational power and data-driven analyses enabled by such technologies.

## Conclusion

AI has immense potential in pediatric allergy, although implementation in research and clinical practice is still at the foundational stage. Improved methodology, increased detail in reporting of computational aspects in studies, and increased collaboration are central to accelerate impactful discoveries. Advanced models are also underutilized, as are multi-omics and rich unstructured data. Finally, decision-makers and the general public (not least patient-representative groups) should be involved in development of AI-based technologies in order to increase awareness of utility, instill trust, and reduce bias of such applications.

## Supplementary Information

Below is the link to the electronic supplementary material.

Supplementary file1 (DOCX 71 KB)

## Data Availability

No datasets were generated or analysed during the current study.

## Abbreviations

*AD*: Atopic dermatitis
*AI*: Artificial intelligence
*ANN*: Artificial neural network
*AR*: Allergic rhinitis
*DL*: Deep learning
*DNN*: Deep neural network
*ML*: Machine learning
