# Supplementary material for: Artificial intelligence in pediatric allergy research
Source: Eur J Pediatr. 2024 Dec 21;184(1):98. doi: 10.1007/s00431-024-05925-5 (PMC11662037; doi:10.1007/s00431-024-05925-5)
Supplement: Supplementary file 1 — Supplementary file1 (DOCX 71 KB) [file 431_2024_5925_MOESM1_ESM.docx]

Supplementary material to

**Artificial intelligence in pediatric allergy research**

**Daniil Lisik, Rani Basna, Tai Dinh, Christian Hennig, Syed Ahmar Shah, Göran Wennergren, Emma Goksör, Bright I. Nwaru**

# Table S1. Additional machine learning models

| **Model name^a^** | **Appropriate use-cases / assumptions of data** | | **Advantages/strengths** | **Disadvantages/limitations** | **Examples in pediatric allergy / further reading** |
| --- | --- | --- | --- | --- | --- |
| ***Unsupervised learning*** | | | | | |
| ***k*-modes** / soft rounding *k-*modes [1] etc | | - Cross-sectional categorical data | - Implementations available in most statistical software applications and programming languages - Although base implementation has a potentially suboptimal initialization technique, various iterations are available improving this aspect [2] | - Not suitable for continuous/mixed data - Potential convergence rate issues [1] | *N/A* |
| ***k*-prototypes** / locality sensitive hashing (LSH)-*k*-prototypes etc | | - Cross-sectional mixed (categorical and continuous) data | - Can accommodate mixed data, which is often present in clinical contexts - Computationally efficient [3] | - Performance may reduce in case of non-informative continuous variables [3] | - Clusters of contributing socioeconomic/environmental factors of asthma exacerbations [4] |
| **Gaussian mixture models** | | - Cross-sectional continuous data - Assumed Gaussian distribution within clusters - Allows for flexible within-cluster covariance structure | - Informative by providing probability of assignment (soft clustering) - Allows for statistically principled approach to estimate number of clusters | - Relies on Gaussian assumption within clusters - Non-Gaussian clusters will often be split up into several approximately Gaussian subclusters [5] | - Cytokine expression patterns in exposure to house dust mite [6] |
| **Graph-based clustering** | | - Datasets structured as networks composed of nodes interconnected with edges based on relationships | - Allows for detailed mapping of complex interactions that define hypersensitivity reactions in data - Adapts to various linkage types, preserving the natural structure of biological data | - Computationally intensive, particularly with large datasets involving multiple interactions - Requires precise graph construction to accurately reflect biological complexities | *N/A* |
| **HDBSCAN** | | - Cross-sectional data - Between-cluster separation preferred over within-cluster homogeneity | - Handles outliers and noise effectively - Produces a hierarchy of clusters for exploration at different levels - Scalable for large datasets - Can find clusters with flexible irregular shapes and nonlinear separation | - Sensitive to hyperparameters and may require tuning - Can produce fragmented clusters in uniform data - May produce heterogeneous clusters | *N/A* |
| **Spectral clustering** | | - Cross-sectional continuous data - Focus on between-cluster separation rather than within-cluster homogeneity | - Can identify clusters with irregular shapes and complex nonlinear separation | - High computational cost for large datasets due to the need to compute eigenvectors - Sensitive to the choice of similarity graph and scale parameter - May produce heterogeneous clusters | Phenotypes of asthma based on medical history, anthropometric, lung function, and peripheral blood measures [7]  Endotypes of asthma based on cytokine profiles [8] |
| **Non-negative matrix factorization (NMF)** | | - Tabular non-negative data in matrix form (observations as rows and variables as columns) - Image analysis, speech recognition and language processing | - Possibility to identify overlapping/hierarchical substructures in a more adaptive manner than with e.g., hierarchical clustering [9, 10] | - Relatively computationally expensive, with potentially slow convergence [9] | Longitudinal phenotypes of eczema, asthma, and sensitization [11] |
| **Self-organizing map (SOM)** *(typically used in tandem with subsequent HCA)* | | - Cross-sectional continuous data (generalizations to accommodate categorical data are available [12]) | - Computationally efficient [13] - Useful for visualization of data [14] | - Inconsistent performance on small datasets [15] | *N/A* |
| **Longitudinal *k*-means** | | - Longitudinal continuous data | - Computationally efficient non-model-based alternative for longitudinal analyses [16] | - Not suitable for categorical/mixed data - Subpar performance compared with e.g., growth mixture modeling (GMM) [17] | - Trajectories of asthma, dermatitis, and rhinitis [18] |
| **Deep embedded clustering (DEC) [19]** / X-DEC [20], IDEC [21] etc | | - Cross-sectional data (DEC is primarily applicable on continuous data, although iterations such as X-DEC [20] accommodate mixed data) | - First well-established deep clustering algorithm with well-established performance and multiple iterations, such as IDEC [21]) | - Demands relatively large data for optimal performance | *N/A* |
| **DeepTLF [22]** | | - Cross-sectional mixed data - Can be used in supervised learning as well | - High performant for complex, heterogeneous data | - Decision tree component may have inferior performance in comparison to e.g., an optimized neural transformation layer | *N/A* |
| **Ensemble clustering** | | - Heterogeneous or complex datasets | - Can improve cluster quality by aggregating diverse clustering results | - Computationally intensive due to multiple clustering executions - Complex to implement and tune - Compromising between diverse results from different and potentially inconsistent approaches is not necessarily beneficial | *N/A* |
| ***Supervised learning*** | | | | | |
| **LightGBM** | - Classification or regression tasks - Continuous and categorical data | | - Improving accuracy through ensemble of weak prediction models - Similar performance as XGBoost but more computationally efficient [23] | - Performance issues may arise in high-dimensional data | *N/A* |
| **CatBoost** | - Classification or regression tasks - Categorical, continuous, text data | | - Performs well on complex data including categorical (high cardinality) variables [24] - Require relatively little training data [25] - Accommodates missing data and outliers well [26] | - Performance issues may arise in high-dimensional data [26] - Slow training [26, 27] | - Prediction of asthma diagnosis in inpatient setting [28] |
| **Learning Using Concave and Convex Kernels (LUCCK) [29]** | - Multi-modal data - Suitable in data with large measurement errors [30] | | - Likely suitable in many clinical contexts where measurement error of individual variables is substantial and stemming from multiple modalities [31, 32] | - Relatively novel method and thus not widely available implementations or independent assessments of general performance | - Prediction of OFC outcome [31, 32] |
| **DeepTLF [22]** | - Cross-sectional mixed data - Can be used in unsupervised learning as well | | - High performant for complex, heterogeneous data | - Decision tree component may have inferior performance in comparison to e.g., an optimized neural transformation layer | *N/A* |
| **Super learners /** **ensemble learning [33]** | *Depends on incorporated models* | | - Reduces the risk of overfitting [34] | - Computationally demanding [35] | *N/A* |

The list is not intended to be comprehensive or cover all relevant/possible use-cases, but rather to provide an overview of common and promising algorithms. **Abbreviations.** AD: atopic dermatitis. AI: artificial intelligence. AR: allergic rhinitis. FA: food allergy. FeNO: fraction of exhaled nitric oxide. N/A: not available. OFC: oral food challenge. SNP: single nucleotide polymorphism.

# Supplementary references

1. Gavva ST, Karthik CS, Punna S. Clustering categorical data: Soft rounding k-modes. Information and Computation. 2024;296:105115.

2. Sajidha SA, Chodnekar SP, Desikan K. Initial seed selection for K-modes clustering – A distance and density based approach. Journal of King Saud University - Computer and Information Sciences. 2021;33:693-701.

3. Preud'homme G, Duarte K, Dalleau K, Lacomblez C, Bresso E, Smaïl-Tabbone M, et al. Head-to-head comparison of clustering methods for heterogeneous data: a simulation-driven benchmark. Sci Rep. 2021;11:4202.

4. Khan S, Bajwa S, Brahmbhatt D, Lovinsky-Desir S, Sheffield PE, Stingone JA, Li S. Multi-Level Socioenvironmental Contributors to Childhood Asthma in New York City: a Cluster Analysis. J Urban Health. 2021;98:700-10.

5. Kasa SR, Rajan V. Avoiding inferior clusterings with misspecified Gaussian mixture models. Sci Rep. 2023;13:19164.

6. Wu J, Prosperi MC, Simpson A, Hollams EM, Sly PD, Custovic A, Holt PG. Relationship between cytokine expression patterns and clinical outcomes: two population-based birth cohorts. Clin Exp Allergy. 2015;45:1801-11.

7. Howrylak JA, Fuhlbrigge AL, Strunk RC, Zeiger RS, Weiss ST, Raby BA. Classification of childhood asthma phenotypes and long-term clinical responses to inhaled anti-inflammatory medications. J Allergy Clin Immunol. 2014;133:1289-300, 300.e1-12.

8. Cottrill KA, Rad MG, Ripple MJ, Stephenson ST, Mohammad AF, Tidwell M, et al. Cluster analysis of plasma cytokines identifies two unique endotypes of children with asthma in the pediatric intensive care unit. Sci Rep. 2023;13:3521.

9. Devarajan K. Nonnegative matrix factorization: an analytical and interpretive tool in computational biology. PLoS Comput Biol. 2008;4:e1000029.

10. Gaujoux R, Seoighe C. A flexible R package for nonnegative matrix factorization. BMC Bioinformatics. 2010;11:367.

11. Schoos AM, Chawes BL, Rasmussen MA, Bloch J, Bønnelykke K, Bisgaard H. Atopic endotype in childhood. J Allergy Clin Immunol. 2016;137:844-51.e.

12. del Coso C, Fustes D, Dafonte C, Nóvoa FJ, Rodríguez-Pedreira JM, Arcay B. Mixing numerical and categorical data in a Self-Organizing Map by means of frequency neurons. Applied Soft Computing. 2015;36:246-54.

13. Herrero J, Dopazo J. Combining hierarchical clustering and self-organizing maps for exploratory analysis of gene expression patterns. J Proteome Res. 2002;1:467-70.

14. Dalton L, Ballarin V, Brun M. Clustering algorithms: on learning, validation, performance, and applications to genomics. Curr Genomics. 2009;10:430-45.

15. Coombes CE, Liu X, Abrams ZB, Coombes KR, Brock G. Simulation-derived best practices for clustering clinical data. J Biomed Inform. 2021;118:103788.

16. Mullin S, Zola J, Lee R, Hu J, MacKenzie B, Brickman A, et al. Longitudinal K-means approaches to clustering and analyzing EHR opioid use trajectories for clinical subtypes. J Biomed Inform. 2021;122:103889.

17. Den Teuling NGP, Pauws SC, van den Heuvel ER. A comparison of methods for clustering longitudinal data with slowly changing trends. Communications in Statistics - Simulation and Computation. 2023;52:621-48.

18. Kilanowski A, Thiering E, Wang G, Kumar A, Kress S, Flexeder C, et al. Allergic disease trajectories up to adolescence: Characteristics, early-life, and genetic determinants. Allergy. 2023;78:836-50.

19. Xie J, Girshick R, Farhadi A, editors. Unsupervised deep embedding for clustering analysis. International conference on machine learning; 2016: PMLR.

20. de Kok JWTM, van Rosmalen F, Koeze J, Keus F, van Kuijk SMJ, Castela Forte J, et al. Deep embedded clustering generalisability and adaptation for integrating mixed datatypes: two critical care cohorts. Scientific Reports. 2024;14:1045.

21. Guo X, Gao L, Liu X, Yin J, editors. Improved deep embedded clustering with local structure preservation. Ijcai; 2017.

22. Borisov V, Broelemann K, Kasneci E, Kasneci G. DeepTLF: robust deep neural networks for heterogeneous tabular data. International Journal of Data Science and Analytics. 2023;16:85-100.

23. van Breugel M, Fehrmann RSN, Bügel M, Rezwan FI, Holloway JW, Nawijn MC, et al. Current state and prospects of artificial intelligence in allergy. Allergy. 2023;78:2623-43.

24. Hancock JT, Khoshgoftaar TM. CatBoost for big data: an interdisciplinary review. J Big Data. 2020;7:94.

25. Safaei N, Safaei B, Seyedekrami S, Talafidaryani M, Masoud A, Wang S, et al. E-CatBoost: An efficient machine learning framework for predicting ICU mortality using the eICU Collaborative Research Database. PLoS One. 2022;17:e0262895.

26. Ahn JM, Kim J, Kim K. Ensemble Machine Learning of Gradient Boosting (XGBoost, LightGBM, CatBoost) and Attention-Based CNN-LSTM for Harmful Algal Blooms Forecasting. Toxins (Basel). 2023;15.

27. Saltik Ö, Rehman WU, Kaymaz T, Degirmen S. Herding towards pygmalion: Examining the cultural dimension of market and bank based systems. Heliyon. 2024;10.

28. Yu G, Li Z, Li S, Liu J, Sun M, Liu X, et al. The role of artificial intelligence in identifying asthma in pediatric inpatient setting. Ann Transl Med. 2020;8:1367.

29. Sabeti E, Gryak J, Derksen H, Biwer C, Ansari S, Isenstein H, et al. Learning Using Concave and Convex Kernels: Applications in Predicting Quality of Sleep and Level of Fatigue in Fibromyalgia. Entropy [Internet]. 2019; 21(5).

30. Hernandez L, Kim R, Tokcan N, Derksen H, Biesterveld BE, Croteau A, et al. Multimodal tensor-based method for integrative and continuous patient monitoring during postoperative cardiac care. Artificial Intelligence in Medicine. 2021;113:102032.

31. Zhang J, Lee D, Jungles K, Shaltis D, Najarian K, Ravikumar R, et al. Prediction of oral food challenge outcomes via ensemble learning. Informatics in Medicine Unlocked. 2023;36:101142.

32. Gryak J, Georgievska A, Zhang J, Najarian K, Ravikumar R, Sanders G, Schuler CF. Prediction of pediatric peanut oral food challenge outcomes using machine learning. Journal of Allergy and Clinical Immunology: Global. 2024;3:100252.

33. Mahajan P, Uddin S, Hajati F, Moni MA. Ensemble Learning for Disease Prediction: A Review. Healthcare (Basel). 2023;11.

34. Bartlett P, Freund Y, Lee WS, Schapire RE. Boosting the margin: A new explanation for the effectiveness of voting methods. The annals of statistics. 1998;26:1651-86.

35. Jiang T, Gradus JL, Rosellini AJ. Supervised Machine Learning: A Brief Primer. Behav Ther. 2020;51:675-87.
